# NanoScope wrist arthroscopy under wide-awake local anesthesia with no tourniquet: A prospective series of 30 consecutive patients

**DOI:** 10.1016/j.jham.2024.100067

**Published:** 2024-04-02

**Authors:** Daniel Reiser, Mattias Hedspång, Marcus Sagerfors

**Affiliations:** Department of Hand Surgery and Orthopedics, Faculty of Medicine and Health, Örebro University, SE 70182, Örebro, Sweden

**Keywords:** Wrist, Arthroscopy, WALANT, NanoScope, MRI

## Abstract

**Introduction:**

Wrist arthroscopy is an evolving procedure. The purpose of this study was to report the outcome of diagnostic arthroscopy of the wrist using a new tool, the NanoScope, under wide-awake local anesthesia with no tourniquet (WALANT).

**Patients and methods:**

This was a prospective study of 30 consecutive patients with suspected ligament tear after wrist trauma and remaining symptoms after initial conservative management. All patients had an MRI prior to the NanoScope procedure.

**Results:**

The patients comprised 17 men and 13 women, with a mean age of 31 years. One patient declined the NanoScope procedure following their MRI. In the remaining 29 patients, NanoScope wrist arthroscopy revealed 19 cases of triangular fibrocartilaginous complex (TFCC) tears and 11 tears of the scapholunate (SL) or lunotriquetral (LT) ligaments. The correlation between preoperative MRI and the findings from NanoScope arthroscopy was poor. Six patients had additional surgery after the NanoScope arthroscopy, comprising three TFCC sutures, one SL and one LT ligament reconstruction respectively, and one wrist arthrodesis. No complications related to the NanoScope arthroscopies were noted.

**Conclusion:**

NanoScope arthroscopy of the wrist is safe, is well-suited for surgery in WALANT, and has superior diagnostic capacity compared to MRI. Further studies are warranted to determine the role of the NanoScope in the management of wrist ligament pathologies.

**Level of evidence:**

This is a level 4 study.

## 1 Introduction

It is well known that trauma to the wrist, such as hyperextension, distortion, or a distal radius fracture, poses a risk of ligament tears in the wrist.^1^ Missed ligament tears can lead to an increased risk of osteoarthritis or cartilage wear, resulting in disabling hand and wrist problems.^2^ The diagnosis and correct staging of possible ligament tears is therefore of great importance in order to optimize the prognosis in the individual patient and reduce the risk of later complications.^3^ Treatment varies from immobilization with a supporting splint and hand therapy to surgical treatment,^1^^,^^2^^,^^4^ meaning that not only the mapping but also the staging of the tears is important.^5^

Plain radiographs, ultrasound, and computed tomography may aid in the initial workup of these patients. Although new generations of MRI have better diagnostic ability, arthroscopy is still the gold standard for correct evaluation and classification in the diagnosis of ligamentous disorders of the wrist.^6^, ^7^, ^8^, ^9^, ^10^, ^11^, ^12^, ^13^, ^14^ However, standard arthroscopy using a rigid small joint arthroscope has a risk of iatrogenic injury to the cartilage at the time of entry, especially in small, tight joints. Regular wrist arthroscopy can be equipment-intensive, involving irrigation systems and general anesthesia. The introduction of dry arthroscopy has reduced this issue.^15^ Some patients may undergo arthroscopy surgery where a ligament injury is diagnosed but no further surgical intervention can be recommended. The COVID-19 pandemic resulted in decreased resources for elective surgery with anesthesia in many countries, prompting an increase in procedures performed under local anesthesia.^16^^,^^17^

**Fig. 1:**
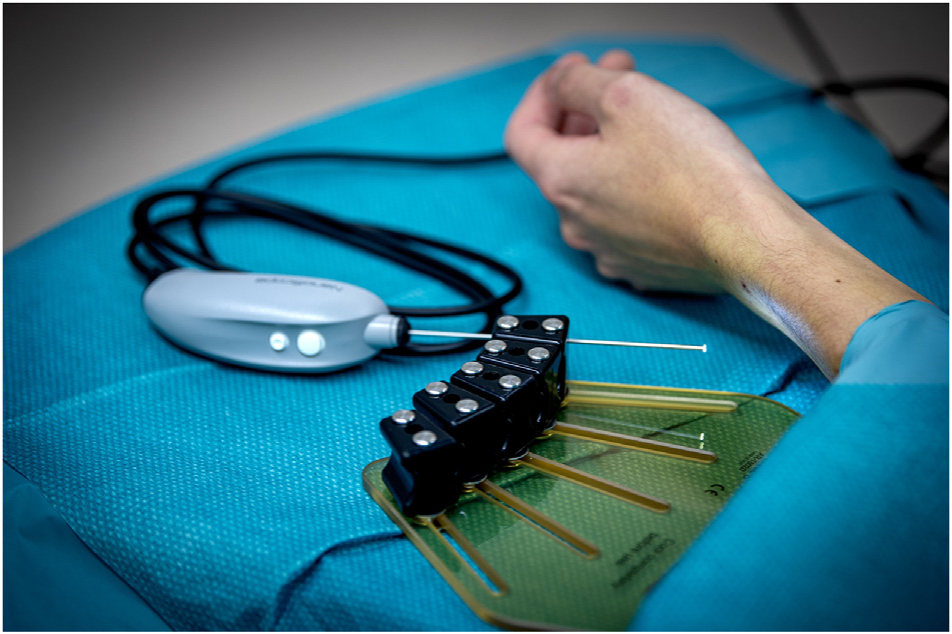
The NanoScope.

**Fig. 2:**
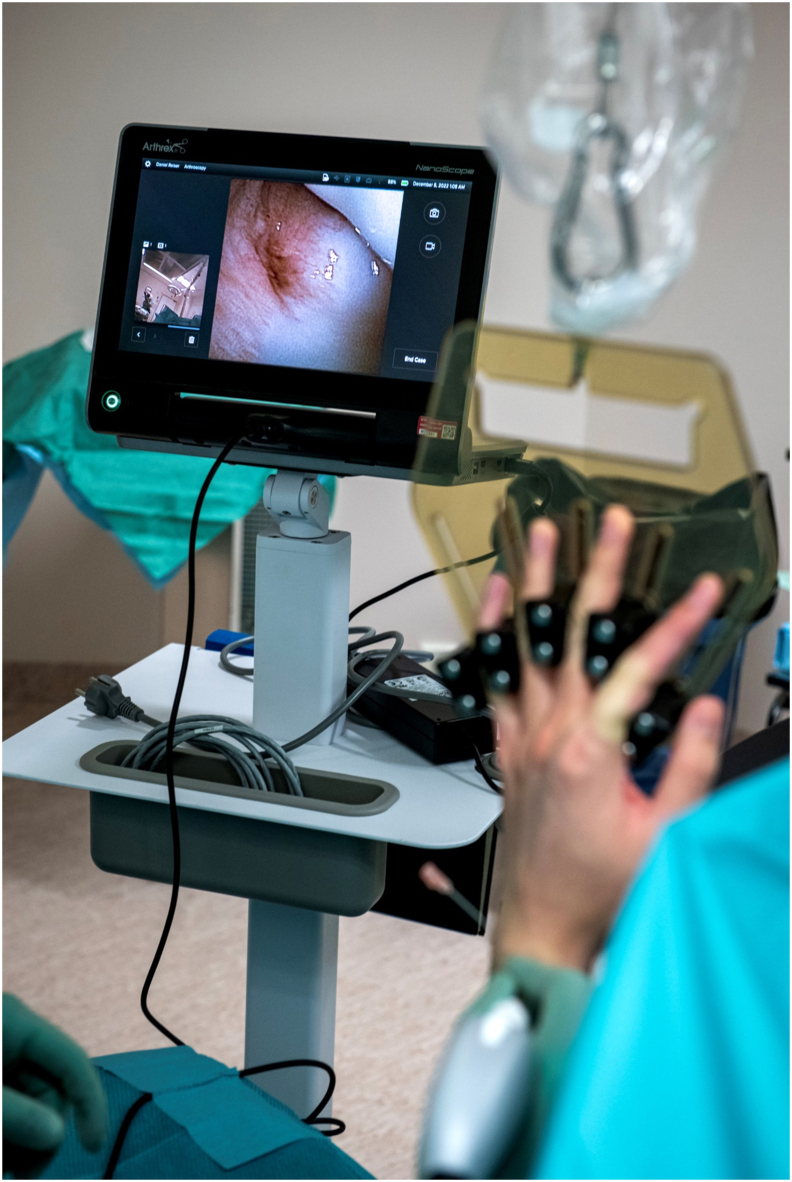
Assessment of findings, portable screen.

The NanoScope (Arthrex, Naples, FL) is a new instrument for arthroscopy with a diameter of 1.9 ​mm. Utilizing a so called “chip-on-tip” technology, the instrument has a 120° panoramic field of view, looking straight on (Fig. 1). The system is for single use, and is attached to a portable screen (Fig. 2). A recent cadaveric study indicated that the system is safe and feasible for wrist arthroscopy.^18^ To our knowledge, clinical studies on the NanoScope in the assessment of wrist pathology are sparse and prospective studies are lacking.^19^^,^^20^

The aim of this study was to report the diagnostic ability of NanoScope arthroscopy of the wrist performed under wide-awake local anesthesia with no tourniquet (WALANT) compared to preoperative MRI findings in a prospective series of 30 consecutive patients. Another aim was to report the safety and reliability of the procedure. Costs for the procedures were also analyzed.

## 2 Materials and methods

The study was approved by the Swedish ethical review authority (reference number: 2021-00469) and was registered in the Swedish Public Trials Registry (FoU i Sverige; https://www.researchweb.org/is/sverige; registration number: 275661). The study was undertaken at the university hospital in Örebro, Sweden, which is a tertiary referral center. Thirty consecutive patients were included, all of whom gave their informed consent according to the Helsinki declaration. The patients had a mean age of 31 years (range: 16–48 years), and comprised 17 men and 13 women. Patients were referred to the hand surgery department with a suspected ligament injury in the wrist. Criteria for participation included age up to 50 years, extension or flexion trauma to the hand/wrist with no improvement after 4–6 weeks, and clinical status findings indicating suspected wrist ligament injury with no osteoarthritis or malalignment on plain radiographs. Assessment of clinical stability was done using a standard clinical wrist examination including Watson's test, Reagan's shuck test, ulnar fovea sign etc.^21^^,^^22^ Patients with reduced autonomy, inability to understand Swedish, or a clinically verified instability in the distal radio-ulnar joint (DRU-J) or in the carpus were excluded. The reason for excluding patients with a clinically verified instability was that these often require ligament reconstruction with open or arthroscopic technique. The longer operation time and more extensive surgery is not well suited for surgery under WALANT, and is conducted under general anesthesia or brachial block instead. All patients underwent MRI of the wrist before the NanoScope procedure (3.0 ​T Discovery™ 750W GE Health Care).

Initial treatment was conservative in all patients, most often with a cast for 4 weeks to immobilize the wrist. If there was no improvement after 4–6 weeks, the patients were included in the study after receiving oral and written information from the attending physician. A MRI was performed in all patients before the NanoScope arthroscopy. After inclusion in the study, the NanoScope arthroscopy was performed within 1–2 weeks, which was a maximum of 12 weeks after the trauma. The surgery was performed in the operating theater as a dry arthroscopy by one of two hand surgeons with experience level 3–4 according to Tang.^23^ Preoperatively, 20 ​ml mepivacaine with epinephrine with or without buffers was administered in the different standard portals to the level of the joint capsule plus intra-articular infiltration. A 30 ​min delay was planned to optimize the hemostatic effect of the adrenaline.

**Fig. 3:**
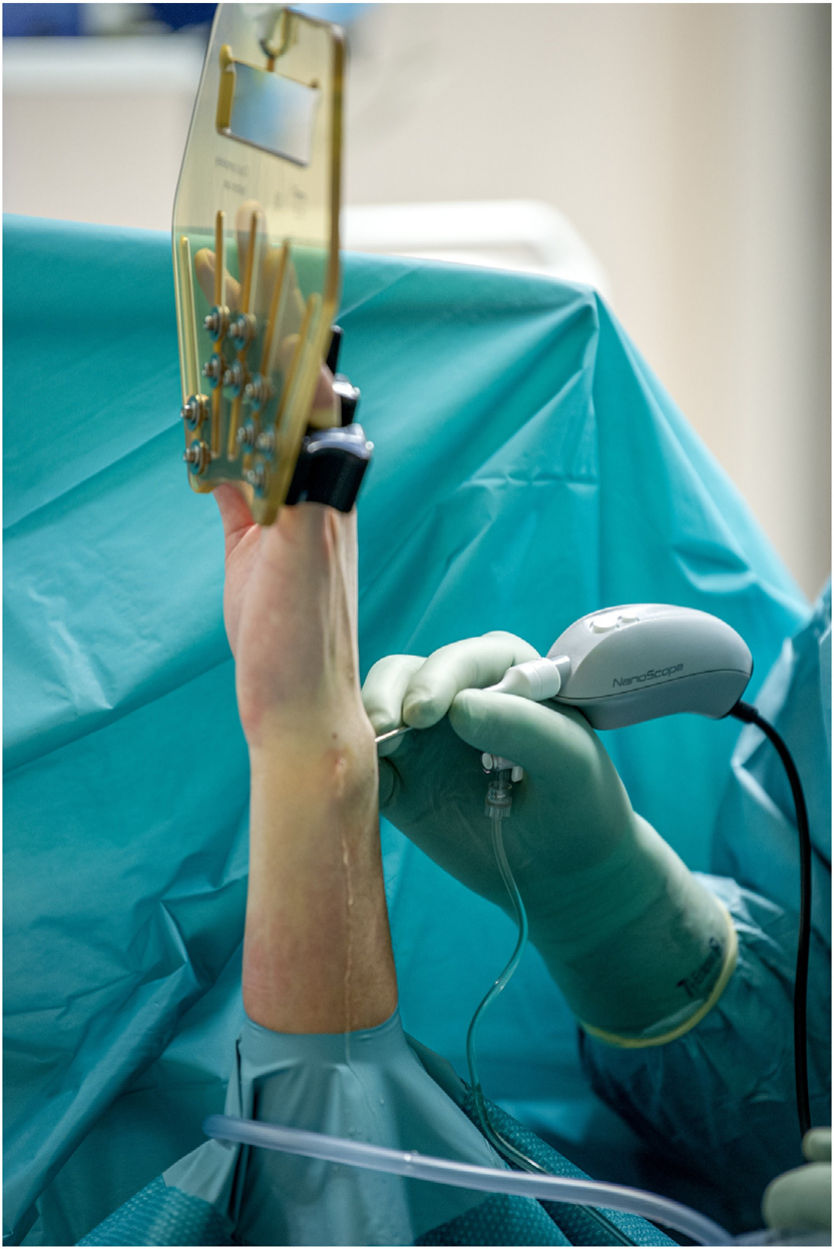
Wrist flushed with saline during shaving.

The patient was placed in a standard wrist arthroscopic setup with 4 ​kg traction (Wrist Traction Tower, Arthrex GmbH). As a precaution, a pneumatic tourniquet was applied but was never inflated. After checking for adequate analgesia, a 3 to 4 portal was established. Midcarpal and 6R portals were established, but as the camera views end on with no angulation there was some variation in placement, which is a deviation from a standard arthroscopy.^20^ The sharp chip-on-tip system obturator was used like a needle to penetrate the skin. Ports were changed as needed, because the needle chip-on-tip system is flexible and if moved sideways against resistance it will flex. The wrist was examined and the findings evaluated (Fig. 3). Triangular fibrocartilaginous complex (TFCC) tears were classified according to Atzei, and scapholunate (SL) or lunotriquetral (LT) tears were classified according to Geissler.^24^^,^^25^ An arthroscopic synovectomy (Arthrex Shaver 2.3 ​mm) ensured full visualization as a therapeutic measure if necessary. During shaving, the wrist was also flushed with saline solution through the portal. After finishing the procedure, the portals were dressed with sterile adhesive dressing; no stitches were required. All patients had a follow-up visit 3 months postoperatively.

Patients who were found to have a ligament injury that required a direct open reconstruction were operated under general anesthesia or brachial block in a second session at a later time.

## 3 Results

All 30 patients underwent a MRI preoperatively. One patient decided not to undergo a NanoScope arthroscopy after the MRI, and so 29 NanoScope arthroscopies were performed. Average time for the procedure was 15 ​min. Wrist arthroscopy with the NanoScope revealed 11 cases with Atzei 1 tears, one in combination with a central tear; four cases with Atzei 2 tears, two in combination with a central tear; three Atzei 3 tears; and one Atzei 4 tear. In the remaining 10 cases, the TFCC was without a tear. MRI showed five tears of the triangular fibrocartilage complex (TFCC) in the 30 cases. In addition, MRI showed 10 cases of SL ligament tears, only four of which could be confirmed arthroscopically (Table 1 and 2a) (See Table 2b).

**Table 1:** Types of tears classified according to Atzei and Geissler.

Tear	Arthroscopy
TFCC Atzei 1	10
TFCC Atzei 1+4	1
TFCC Atzei 2	2
TFCC Atzei 3	3
TFCC Atzei 2+4	2
TFCC Atzei 4	1
**Total**	19
SL Geissler 1	1
SL Geissler 2–3	3
SL Geissler 4	4
LT Geissler 2–3	2
LT Geissler 4	1
**Total**	11

LT ​= ​lunotriquetral ligament tear, SL ​= ​scapholunate ligament tear, TFCC ​= ​triangular fibrocartilaginous complex tear.

**Table 2a:** Correlation between MRI findings and findings during NanoScope wrist arthroscopy. Carpal ligament tears include both scapholunate ligament and lunotriquetral ligament tears.

MRI total	30
MRI carpal tears correct diagnosis	3
MRI carpal tears false negative diagnosis	6
MRI carpal tears false positive diagnosis	7
MRI TFCC tears correct diagnosis	4
MRI TFCC tear false negative diagnosis	15
MRI TFCC tear false positive diagnosis	1

TFCC ​= ​triangular fibrocartilaginous complex.

**Table 2b:** Sensitivity and specificity of MRI findings regarding carpal plus TFCC tears in comparison with findings during NanoScopy.

	NanoScopy	NanoScopy
MRI positive	True positive 7	False positive 8
MRI negative	False negative 21	True negative 6
	Sensitivity 25%	Specificity 43%

Several patients showed different combinations of ligament tears, both carpal (SL plus LT) and in the TFCC, during NanoScope arthroscopy (Table 3). Acute ligament tears in the carpus were treated with immobilization in a plaster splint and subsequent rehabilitation with a hand therapist. TFCC injuries affecting the stability of the DRU joint (Atzei 2 and 3) received special wrist stability training from a hand therapist over a period of 3 months. Patients with unsatisfactory results (mainly pain and instability symptoms) after conservative therapy were operated under anesthesia with ligament suture or reconstructions. A total of three TFCC sutures, one LT reconstruction, and one SL reconstruction were performed. One patient presented with cartilage degeneration found during the NanoScope arthroscopy but not readily visible on plain radiographs. After unsatisfactory treatment effect with cortisone injection, activity modification, and a wrist splint, a decision was taken to proceed with radiocarpal arthrodesis due to pain and a need to sustain heavy load to the wrist. No complications related to the NanoScope procedure were noted. No further sedation or anesthesia was needed during the NanoScope procedure.

**Table 3:** Findings during NanoScope wrist arthroscopy and treatment.

NanoScope findings	LT tear	SL tear	Number of cases	Primary treatment	Result
TFCC Atzei 1	–	–	6	Rehabilitation	Satisfactory wrist function
TFCC Atzei 1	–	Geissler 3	1	Cast and Rehabilitation	Satisfactory wrist function
TFCC Atzei 1	–	Geissler 4	2	Cast and Rehabilitation	1 SL reconstruction
TFCC Atzei 1	Geissler 4	–	1	Cast and Rehabilitation	1 LT reconstruction
TFCC Atzei 1+4	–	Geissler 2	1	Rehabilitation	Satisfactory wrist function
TFCC Atzei 2	–	–	2	Rehabilitation	1 TFCC suture
TFCC Atzei 2+4	–	–	2	Rehabilitation	2 TFCC suture
TFCC Atzei 3	–	–	2	Rehabilitation	Satisfactory wrist function
TFCC Atzei 3	–	Geissler 2	1	Rehabilitation	Satisfactory wrist function
TFCC Atzei 4 and arthrosis	–	–	1	Orthosis	Radiocarpal fusion
No TFCC tear	–	Geissler 1	1	Cast and Rehabilitation	Satisfactory wrist function
No TFCC tear	–	Geissler 4	2	Cast and Rehabilitation	Satisfactory wrist function
No TFCC tear	Geissler 3	–	1	Cast and Rehabilitation	Satisfactory wrist function

LT ​= ​lunotriquetral ligament, SL ​= ​scapholunate ligament, TFCC ​= ​triangular fibrocartilaginous complex.

The cost of the NanoScope arthroscopy carried out under WALANT in the operating theatre was 8000 Swedish krona (SEK), which at the time of writing corresponds to around 780 US dollars (USD). At our hospital, the average standard cost for a diagnostic arthroscopy performed under anesthesia in the regular operating theatre is 50,000 SEK (about 4860 USD), and the cost for a MRI is 4868 SEK (438 USD) (Table 4).

**Table 4:** Advantages and disadvantages of Nanoscope wrist arthroscopy in WALANT.

Advantage of NanoScop in WALANT	Disadvantage of NanoScop in WALANT
No need for a regular arthroscopy tower	No angulation of the chip-on tip system, makes it harder to look up/down
Superior diagnostic ability compared to MRI	More expensive than an MRI
Procedure done in WALANT, no need for anesthesia resources	No capacity to proceed with a reconstructive procedure if noted during NanoScope arthroscopy
Reduced cost compared to a regular wrist arthroscopy	Not suited for long reconstructive procedures
Arthroscopic synovectomy possible	
Minimally invasive	
Ability to directly inform patient about intraoperative findings, shared decision-making and planning	

## 4 Discussion

We use the NanoScope as a real-time diagnostic alternative to MRI to quickly establish a diagnosis and plan the treatment. As is known from previous studies, arthroscopy is the gold standard for the correct diagnosis of ligament tears in the wrist.^7^, ^8^, ^9^ The overall poor correlation in our study between MRI and findings during NanoScope arthroscopy confirms this.

The literature contains differing opinions on the correct treatment of carpal and TFCC ligament tears, but the importance of a correct diagnosis is undisputed. It is of paramount importance not only to diagnose the ligament tear, but also to stage and classify the tear correctly in order to guide the treatment.^24^^,^^26^ In our experience, Geissler 1 tears can often be successfully treated conservatively if diagnosed in time, and the same applies to Atzei 1 tears of the TFCC complex. We also often address more extensive carpal ligament tears or tears in the TFCC complex primarily with conservative therapy. In this study, clinically clear carpal or DRU instabilities were excluded, and instead went for direct reconstruction; it is important to take this into account when evaluating our treatment results. We performed one SL ligament reconstruction and one LT ligament reconstruction. Both had a Geissler 4 tear. These are difficult to treat conservatively despite intensive stability training and rehabilitation, especially if the patients are blue-collar workers.^27^

The same applies to TFCC tears in the radioulnar ligaments, which can lead to impaired instability in the DRU joint. Three out of seven patients with Atzei 2 or 3 tears did not achieve satisfactory results with conservative treatment. One patient with an Atzei 4 lesion underwent NanoScopy, this should have been identified earlier during the clinical examination. The main problem was pain and instability symptoms when loading the wrist, and they later underwent treatment with regular arthroscopic ligament suture of the TFCC. In these patients we could not diagnose any clinical instability in the DRU joint pre-operatively, which highlights the importance of arthroscopy for a correct diagnosis and subsequent treatment.

We find the NanoScope to be a valuable diagnostic tool in patients with posttraumatic wrist pain and suspected ligament tears. It is well suited for surgery under WALANT, which is an advantage as the COVID-19 crisis has put strain on resources for surgery in general anesthesia.^17^ The fact that only six out of 30 patients needed an additional reconstructive procedure with anesthesia procedure supports this. The cost of the procedure is advantageous compared to a regular wrist arthroscopy in general anesthesia but more expensive than an MRI. More data are required to perform a more in-depth cost analysis of the technique. In addition, cost calculations in a state funded health care system like Sweden can be cumbersome. A wrist MRI has a lower cost, but the diagnostic ability is far from optimal today. The MRIs were assessed by specialists in radiology, but not all were assessed by dedicated musculoskeletal radiologists. This could be one explanation for the poor correlation between MRI and findings during NanoScopy.

Another advantage of the NanoScope is the simplicity of the procedure, as no full arthroscopy tower is required; instead, a portable unit and single-use sterile set with the NanoScope handpiece is sufficient. In our experience, the main difference from a standard arthroscopy is the chip-on-tip handpiece. The chip-on-tip system is flexible, and the lack of angulation makes it harder to look up or down. The system bends when moving sideways, and so our technique has been modified compared to a regular arthroscopy. The movements with the handpiece are reduced, and if better visualization is needed the portal is changed. In our experience, blood in the field obstructing visualization has not been a problem. A previous cadaveric study found one injury to the dorsal sensory branch of the ulnar nerve with the NanoScope.^18^ No complications related to the procedure were noted in our study, which is encouraging. Patient comfort during the procedure was not assessed using a validated outcome instrument and this is a limitation.

The NanoScope thus appears to be a valuable tool in diagnosing ligament tears in the wrist, with superior diagnostic capacity compared to wrist MRI. It also seems to be cost-efficient compared to regular wrist arthroscopy, but a more detailed health economic analysis would probably be beneficial. The procedure is safe, minimally invasive, and well-suited for surgery under WALANT. The NanoScope has been used for diagnostic arthroscopy of the knee in an office setting, this could likely be an option for diagnostic wrist arthroscopies as well.^28^ The Nanoscope can potentially transform diagnostic wrist arthroscopy into an office procedure, thereby further increasing cost-effectiveness. Further studies are warranted to clarify the role of the NanoScope in relation to standard wrist arthroscopy.

## COI statement

The authors have no competing interests.

## Informed consent statement

The study was performed according to the Helsinki declaration and approved by the Swedish Ethical Review Authority (reference number 2021-00469).

## Sources of support

This study was funded by a grant from Örebro County Council (ALF project, grant number: 979910). The funding body had no part in the design of the study, collection, analysis and interpretation of data, or in the writing of the manuscript.
